# Alcohol Reduction to Reduce Relapse in Acute Alcoholic Pancreatitis—Missed Opportunities

**DOI:** 10.1093/alcalc/agab014

**Published:** 2021-03-26

**Authors:** Noor J Sissingh, Devica S Umans, Anna E Goudriaan, Martijn Sijbom, Robert C Verdonk, Jeanin E van Hooft, for the Dutch Pancreatitis Study Group

**Affiliations:** Department of Gastroenterology and Hepatology, Leiden University Medical Center, Pb 9600, 2300 RC, Leiden, The Netherlands; Department of Research and Development, St. Antonius Hospital, Pb 2500, 3430 EM, Nieuwegein, The Netherlands; Department of Research and Development, St. Antonius Hospital, Pb 2500, 3430 EM, Nieuwegein, The Netherlands; Department of Gastroenterology and Hepatology, Amsterdam Gastroenterology and Metabolism, Amsterdam UMC, University of Amsterdam, Pb 22660, 1100 DD, Amsterdam, The Netherlands; Department of Psychiatry, Academic Medical Center, Pb 22660, 1100 DD, Amsterdam, The Netherlands; Department of Public Health and Primary Care, Leiden University Medical Center, Pb 9600, 2300 RC, Leiden, The Netherlands; Department of Gastroenterology and Hepatology, St. Antonius Hospital, Pb 2500, 3430 EM, Nieuwegein, The Netherlands; Department of Gastroenterology and Hepatology, Leiden University Medical Center, Pb 9600, 2300 RC, Leiden, The Netherlands

## Abstract

**Aim:**

Resuming drinking is a main contributant to recurrence in alcoholic pancreatitis. We assessed current clinical practice in the Netherlands regarding alcohol in managing patients with a first episode of acute alcoholic pancreatitis.

**Methods:**

A survey was distributed to 35 hospitals affiliated with the Dutch Pancreatitis Study Group. We evaluated current support based on various components of brief interventions, the participation of psychosocial healthcare providers, the cooperation with the primary care physicians and the presence of a protocol and its implementation.

**Results:**

The response rate was 100% (*n* = 35). Psychoeducation is the most frequently performed intervention in current support treatment (97% of hospitals). In 17% of hospitals, healthcare providers with a psychosocial background routinely participate in current support treatment; 37% of hospitals create an individual treatment plan in which goals regarding alcohol cessation are specified and only 46% of hospitals provide the primary care physician with specific discharge information; 31% of hospitals indicate that the treatment is uniformly performed within their division of Gastroenterology. Protocols are available in 3% of the hospitals surveyed. Opportunities to involve the patient’s social network were not given sufficient priority.

**Conclusion:**

Among Dutch hospitals, there is no routine management strategy with regard to enhancing treatment for heavy alcohol use in alcoholic pancreatitis patients. There is a need to test a validated support program in randomized studies. Meanwhile, possible opportunities for effecting change are often missed.

## INTRODUCTION

Approximately 1300 patients per year with a first episode of acute alcoholic pancreatitis are admitted to hospital in the Netherlands (Dutch Healthcare Authority, 2020; Dutch Hospital Data, 2016). Acute alcoholic pancreatitis can vary from a mild (80% of patients) to a severe and even life-threatening disorder (20% of patients). Severe complications of acute pancreatitis include (infected) pancreatic necrosis and multiple organ failure (Besselink *et al*., 2008a).

A Dutch cross-sectional study has shown that 25% of patients after a first episode of alcoholic pancreatitis have at least one recurrent attack and 16% develop chronic pancreatitis (Ahmed Ali *et al.*, 2016). Continuation of alcohol consumption is the most important modifiable risk factor for the development of this type of recurrent and chronic pancreatitis (Bertilsson *et al.*, 2015; Ahmed Ali *et al.*, 2016). Although cessation of alcohol use can reduce the recurrence rate of alcoholic pancreatitis to near 0% (Pelli *et al.*, 2008; Nikkola *et al.*, 2013), it is notoriously difficult to stop harmful drinking without treatment. Nonetheless, effective treatments—with small to moderate effect size compared to no treatment—are available and include brief interventions (DiClemente *et al.*, 2017). These interventions focus on the patient’s context of harmful drinking, the patient’s motivation to change drinking behavior and on achievable individual treatment goals which might include cessation or reduction of alcohol consumption or change in drinking habits (The Netherlands Psychiatric Association, 2009; Boomsma *et al.*, 2014; Reus *et al.*, 2018).

Unfortunately, international evidence-based guidelines on the treatment of acute alcoholic pancreatitis (Working Group IAP/APA Acute Pancreatitis Guidelines, 2013; Crockett *et al.*, 2018) make no statements on enhancing treatment for heavy alcohol use and its timing (Lawrence *et al.*, 2017). Currently, there is increasing evidence that brief interventions during an admission related to harmful drinking result in significant reduction of alcohol consumption (Alaja and Seppa 2003; McQueen *et al.*, 2011). This effect can be explained by the large impact of hospitalization, making patients motivated for cessation and interventions more successful. Only one randomized controlled trial has studied the effect of a repeated program (at 6 months-interval) with brief interventions to assist in alcohol cessation specifically in patients with alcohol induced pancreatitis. In this study, a 61.9% reduction was observed in pancreatitis recurrence (from 21 to 8%) in patients receiving a repeated intervention when compared with a single session in-hospital intervention (Nordback *et al.*, 2009).

The fact that the incidence of recurrent alcoholic pancreatitis is still 25%, costing ~1.75 million euros yearly, emphasizes the need to develop a validated alcohol cessation support program for patients with alcoholic pancreatitis, in order to reduce recurrence of alcoholic pancreatitis (Andersson *et al.*, 2013; Ahmed Ali *et al.*, 2016). In addition, continuation of (heavy) alcohol use is not only detrimental to the pancreas, it is also associated with a large social burden and the development of cancer and liver, cerebral and cardiovascular diseases (Rehm *et al.*, 2017). Therefore, it is crucial to address alcohol use at the time when patients are receptive to change lifestyle, namely during the clinical admission of acute alcoholic pancreatitis. However, little is known about current support treatment regarding alcohol cessation, both during admission and after discharge, in patients diagnosed with acute alcoholic pancreatitis. To gain insight into current practice and to assess whether improvements can be made, this nationwide survey study was conducted.

## MATERIAL AND METHODS

In June 2020, the Dutch Pancreatitis Study Group (DPSG) designed an online survey with 13 closed questions, as listed in the Supplementary Material. The DPSG is a nationwide study group of clinical experts and researchers involved in pancreatitis care and is known for conducting high-quality research (Bakker *et al.*, 2012, 2014; Besselink *et al.*, 2008a, 2008b; da Costa *et al.*, 2015; Van Brunschot *et al.*, 2018). The survey included questions about current support treatment for a ‘typical’ patient with initial admission for acute alcoholic pancreatitis (mild pancreatitis episode, according to the Revised Atlanta Criteria, with a mean hospital stay of 5 days; Banks *et al.*, 2013). We evaluated current support treatment based on different components of brief interventions (i.e. providing information about the harmful effects of drinking alcohol and the existence of self-help organizations), the type of engaged professionals (i.e. consultation of a psychosocial healthcare provider), the treatment setting (i.e. attendance of patient’s social network), the discharge planning (i.e. communication to a primary care physician) and the presence of a protocol and its implementation. In this survey, psychosocial healthcare providers include medical psychologists and social workers, as in-hospital consultation for psychosocial problems in the Netherlands is most commonly performed by these professionals. The psychologist is able to diagnose and treat alcohol use disorders, whereas the social worker focuses more on practical support and advice. The respondents surveyed were able to answer the survey with ‘yes’, ‘no’ or ‘I do not know’.

The survey was sent by an e-mail to 35 divisions of Gastroenterology in hospitals affiliated with the DPSG, as in the Netherlands, patients with an alcohol induced pancreatitis are almost always admitted to the gastroenterology ward instead of the surgery or (internal medicine ward (Dutch Healthcare Authority, 2020; Dutch Hospital Data, 2016). Two reminders were sent in June and July 2020 to achieve a high response rate.

Data analyses were performed in SPSS and all data are presented in number and percentage for categorical variables (24).

## RESULTS

The survey response rate was 100% (35/35). There was representation from academic hospitals (*n* = 8), training hospitals (*n* = 24) and regional hospitals (*n* = 3).

### Brief intervention during admission

In the vast majority of hospitals (34/35, 97%), the treating physician (i.e. gastroenterologists or residents) informed the patient about the harmful effect of alcohol consumption on the pancreas. Support organizations such as the Alcoholic Anonymous Netherlands were discussed with patients in 30 hospitals (86%). The patient’s social network (i.e. partner, family or friend) was invited to attend the educational consultations between the patient and treating physician in 23 hospitals (66%).

### Involvement of other care providers

During admission, 16 hospitals (46%) called the primary care physician to obtain background information regarding the patient. In six hospitals (17%), a healthcare provider with a psychosocial background (i.e. medical psychologist or social worker) routinely visited the patients for a clinical consultation. In four of these six hospitals, both medical psychologists and social workers met patients with an alcoholic pancreatitis for a face-to-face consultation. In the remaining two hospitals, patient’s consultation was only performed by a medical psychologist. In 11 hospitals (31%) a healthcare provider with a psychosocial background did participate in the support treatment, however face-to-face consultations with patients were not part of standard care. In 18 hospitals (51%), neither the medical psychologists nor the social workers were routinely involved in the support treatment of patients with an acute alcoholic pancreatitis.

### Discharge planning

Before discharge, 13 hospitals (37%) created a treatment plan in which goals with regard to alcohol cessation or reduction were specified in concordance with the patient. A total of 16 hospitals (46%) provided specific advice to the primary care physician for the post-hospital care. In addition to the written discharge summary, oral discharge communication to primary care physicians was given by 13 hospitals (37%).

### Current implementation

In the majority of hospitals (97%), the treatment strategy for patients with alcoholic pancreatitis with regard to alcohol cessation is not structured in a protocol. In total, 13 responders (37%) indicated that the treatment is not uniformly performed within their department of Gastroenterology. Of the remaining responders, 11 (31%) indicated that the treatment is uniformly performed and 11 (31%) indicated that they did not know the answer.

## DISCUSSION

The aim of this nationwide survey was to evaluate current interventions regarding alcohol use in patients diagnosed with a first episode of acute alcoholic pancreatitis. Supportive treatment of alcohol cessation is important, as abstinence or controlled use can prevent recurrence of alcoholic pancreatitis (Pelli *et al.*, 2008; Nikkola *et al.*, 2013). Given that admission for acute alcoholic pancreatitis provides a teachable moment for patients (Alaja and Seppa, 2003), it seems crucial that intervention should start during clinical admission. Despite the fact that gastroenterologists treat many patients with alcohol-related diseases (i.e. pancreatitis, gastrointestinal cancer and liver diseases including cirrhosis), this study demonstrate that (inter)national guidelines on problematic alcohol use are not well implemented (The Netherlands Psychiatric Association, 2009; Boomsma *et al.*, 2014; Reus *et al.*, 2018).

According to these guidelines, brief in-hospital intervention is recommended in all patients with an identified alcohol problem. Brief intervention usually includes psychoeducation and motivational interviewing (MI; The Netherlands Psychiatric Association, 2009). Aspects of psychoeducation are providing education about the harmful effects of drinking alcohol and the existence of self-help organizations. This provides patients with the ability to make informed decisions regarding alcohol cessation (Yeh *et al.*, 2017). We demonstrate that the majority of hospitals already use psychoeducation through education conversations frequently.

MI is the second intervention that should take place, as it is more effective than psychoeducation (Miller *et al.*, 1995). MI is often a brief intervention of a maximum of 30 min that focuses on increasing the patient’s intrinsic motivation, which enhances behavioral change and stimulates seeking for further treatment, for example in the setting of primary care. Previous studies showed that in-hospital motivational interventions are effective in reducing alcohol consumption and this also applies to patients who did not ask for help (Vasilaki *et al.*, 2006). The success of MI depends on the interviewer’s experience and technique and can be improved by training (Broers *et al.*, 2005; Daeppen *et al.*, 2009). In addition, the type of professional providing MI seems to have less influence on the effect of MI (The Health Foundation Inspiring Improvement, 2011), but providing training seems essential as this enhances success. Remarkably, healthcare providers trained for psychosocial interventions and available for consultations in hospitals (i.e. psychologists and social workers) are actively engaged regarding alcohol cessation support only in the minority of responding hospitals. The percentage is even lower when it involves a face-to-face consultation between the psychologist or social worker and the patient (17%). Hence, clinical practice frequently consists of solely treating pancreatitis while omitting brief interventions to treat heavy alcohol use. Although much effort is put into optimizing the treatment of biliary pancreatitis with the aim of preventing recurrent biliary attacks, patients with alcoholic pancreatitis are not provided with a brief intervention including MI for their problematic alcohol use. This results in leaving patients at risk of resuming drinking after discharge and further harm (DiClemente *et al.*, 2017).

Another prerequisite for good support for alcoholic pancreatitis patients is the involvement of patient’s social network (i.e. family, partner or friend), as it increases the treatment success (Sisson and Azrin, 1986; Meyers *et al.*, 2002). Despite this, the social network is invited to attend the (educational and motivational) interventions in 65% of hospitals. Moreover, this study shows that communication from hospitals to primary care physicians can be improved, both during admission and before discharge. The primary care physician has a longstanding relationship with the patient, therefore being aware of other (alcohol-related) health and mental problems, previous alcohol cessation attempts of the patient and the context of the harmful drinking. We show that about half of the hospitals surveyed consult the primary care physician for background information of the patient. To ensure continuity of care in the home phase, primary care physicians should be informed about the admission and reason for admission and what has been discussed with the patient during in-hospital counselling. Finally, it needs to be underlined that primary care physicians are potential key players in alcohol cessation support in the post-hospitalization setting (Oslin *et al.*, 2014). However, we show that primary care physicians receive concrete (usually written) discharge information from only half of the hospitals.

Lack of uniformity in the implementation of support treatment is also indicated by the majority of respondents. In addition, a standardized protocol for the treatment of patients with alcoholic pancreatitis is available in only one hospital. This might imply the need for a standard and validated protocol.

Potential barriers to implement a nationwide alcohol cessation support program on the level of healthcare professionals may include lack of time, lack of training and doubts about its efficacy. To overcome these barriers, well-designed preferably randomized trials, in which positive health benefits of brief interventions are demonstrated, are needed in order to create support for implementation of an alcohol cessation support program. Barriers among patients may include resistance to accept the need for alcohol counseling, as drinking is often socially accepted and most patients do not recognize the harmful effects of alcohol. Organizational barriers may be the multidisciplinary nature of an alcohol cessation support program and that an ‘one-size-fits-all’ program might not be suitable for every patient. Despite all barriers, the fact that in-hospital counseling for alcohol use is part of Dutch insured care can act as a facilitator of implementation.

The strength of this study is the response rate of 100%, which might be a reflection of the importance of this topic felt by gastroenterologists. As we included academic hospitals, teaching hospitals and regional hospitals, our results reflect current clinical practice regarding support for alcohol use in all practice settings, despite the small sample size.

This study also has some limitations. First of all, one limitation is the inclusion of divisions of Gastroenterology with a specific interest in pancreatitis that may be more motivated in treating alcoholic pancreatitis. Therefore, the different aspects of current practice evaluated in our study, such as engaging psychosocial healthcare providers or patient’s family during clinical admission and making discharge phone calls to the primary care physician, may be overestimated. Second, this survey was limited to hospitals from the Netherlands only. As no international literature is available on this topic, insight in the current practice on supportive treatment for problematic alcohol use in alcoholic pancreatitis patients from other countries remains unclear. Third, the population of patients with alcoholic pancreatitis is very heterogeneous. For this reason, it was sometimes difficult for the respondents to answer questions about current practice. In conclusion, this national survey study shows suboptimal implementation of brief interventions for alcohol use and insufficient discharge planning for patients diagnosed with alcoholic pancreatitis. These findings accentuate that improvements for supportive treatment of alcohol use can be made. Future studies should determine whether implementation of a multidisciplinary alcohol cessation support program can reduce pancreatitis recurrence in patients with both mild and severe alcoholic pancreatitis. The results of this survey study can help the design of a prospective randomized trial.

## Supplementary Material

Current_clinical_practice_to_enhance_alcohol_Supp1_agab014
