# Supplementary material for: Alcohol Reduction to Reduce Relapse in Acute Alcoholic Pancreatitis—Missed Opportunities
Source: Alcohol Alcohol. 2021 Mar 26;56(6):678–82. doi: 10.1093/alcalc/agab014 (PMC8686671; doi:10.1093/alcalc/agab014)
Supplement: Current_clinical_practice_to_enhance_alcohol_Supp1_agab014 [file Current_clinical_practice_to_enhance_alcohol_Supp1_agab014.docx]

**Supplementary Material** – survey questions

**Q1. Is discussing the relationship between pancreatitis and alcohol use with the patient a routine treatment step?**

1. Yes
2. No
3. I do not know

**Q2. Is the patient’s social network (family, partner etc.) routinely invited to attend the educational consultations between patient and treating physician?**

1. Yes
2. No
3. I do not know

**Q3. Is the medical psychologist routinely involved in the support treatment during an admission of acute alcoholic pancreatitis?**

1. Yes
2. No
3. I do not know

**Q4. If yes, does the medical psychologist routinely physically visit the patient during admission?**

1. Yes
2. No
3. I do not know

**Q5. Is the social worker routinely involved in the support treatment during an admission of acute alcoholic pancreatitis?**

1. Yes
2. No
3. I do not know

**Q6. If yes, does the social worker routinely physically visit the patient during admission?**

1. Yes
2. No
3. I do not know

**Q7. Is the general practitioner routinely called to obtain background information about the patient?**

1. Yes
2. No
3. I do not know

**Q8. Is discussing support organizations as the Alcoholic Anonymous (AA) Netherlands a routine treatment step?**

1. Yes
2. No
3. I do not know

**Q9. Before discharge, is a treatment plan in which goals regarding alcohol cessation of reduction are specified routinely created in concordance with the patient?**

1. Yes
2. No
3. I do not know

**Q10. In addition to the written discharge summary, is the general practitioner routinely informed by telephone before discharge?**

1. Yes
2. No
3. I do not know

**Q11. Is advice routinely given to the general practitioner with regard to the post-hospital care?**

1. Yes
2. No
3. I do not know

**Q12. Are the treatment steps mentioned above uniformly performed within your department of Gastroenterology?**

1. Yes
2. No
3. I do not know

**Q13. Does your department have a protocol for alcohol support treatment for alcoholic pancreatitis?**

1. Yes
2. No
